# Enhanced Antimicrobial, Cytotoxicity, Larvicidal, and Repellence Activities of Brown Algae, *Cystoseira crinita*-Mediated Green Synthesis of Magnesium Oxide Nanoparticles

**DOI:** 10.3389/fbioe.2022.849921

**Published:** 2022-02-28

**Authors:** Amr Fouda, Ahmed M. Eid, Mohamed Ali Abdel-Rahman, Ehab F. EL-Belely, Mohamed A. Awad, Saad El-Din Hassan, Zarraq E. AL-Faifi, Mohammed F. Hamza

**Affiliations:** ^1^ Botany and Microbiology Department, Faculty of Science, Al-Azhar University, Cairo, Egypt; ^2^ Department of Zoology and Entomology, Faculty of Science, Al-Azhar University, Cairo, Egypt; ^3^ Center for Environment Research and Studies, Jazan University, Jazan, Saudi Arabia; ^4^ School of Nuclear Science and Technology, University of South China, Heng Yang, China; ^5^ Nuclear Materials Authority, Cairo, Egypt

**Keywords:** Brown algae, *Cystoseira crinita*, green synthesis, MgO-NPs, antimicrobial, *in-vitro* cytotoxicity, larvicidal, repellence activity

## Abstract

Herein, the metabolites secreted by brown algae, *Cystoseira crinita*, were used as biocatalyst for green synthesis of magnesium oxide nanoparticles (MgO-NPs). The fabricated MgO-NPs were characterized using UV-vis spectroscopy, Fourier transforms infrared spectroscopy (FT-IR), Transmission Electron Microscopy (TEM), Scanning Electron Microscopy linked with energy-dispersive X-ray (SEM-EDX), X-ray diffraction (XRD), and X-ray photoelectron spectroscopy (XPS). Data showed successful formation of crystallographic and spherical MgO-NPs with sizes of 3–18 nm at a maximum surface plasmon resonance of 320 nm. Moreover, EDX analysis confirms the presence of Mg and O in the sample with weight percentages of 54.1% and 20.6%, respectively. Phyco-fabricated MgO-NPs showed promising activities against Gram-positive bacteria, Gram-negative bacteria, and *Candida albicans* with MIC values ranging between 12.5 and 50 μg mL^−1^. The IC_50_ value of MgO-NPs against cancer cell lines (Caco-2) was 113.4 μg mL^−1^, whereas it was 141.2 μg mL^−1^ for normal cell lines (Vero cell). Interestingly, the green synthesized MgO-NPs exhibited significant larvicidal and pupicidal activity against *Musca domestica.* At 10 μg mL^−1^ MgO-NPs, the highest mortality percentages were 99.0%, 95.0%, 92.2%, and 81.0% for I, II, III instars’ larvae, and pupa of *M. domestica*, respectively, with LC_50_ values (3.08, 3.49, and 4.46 μg mL^−1^), and LC_90_ values (7.46, 8.89, and 10.43 μg mL^−1^), respectively. Also, MgO-NPs showed repellence activity for adults of *M. domestica* at 10 μg mL^−1^ with 63.0%, 77.9%, 84.9%, and 96.8% after 12, 24, 48, and 72 h, respectively.

## Introduction

Nanomaterials are materials with at least one dimension and an average size of 1–100 nm with an extraordinary surface area. Its fabrication conditions, shape, and size can be precisely controlled to exhibit certain mechanical, electrical, magnetic, and optical catalytic properties, which distinguish them from their bulk material counterparts (Baig et al., 2021; Shaheen et al., 2021). Magnesium oxide nanoparticles (MgO-NPs) are present in diverse morphological frameworks such as needles, rods, platelets, cubes, flowers, spheres, and stars that qualify them to produce novel nanomaterials (Cao et al., 2020). Currently, green synthesis methods for nanoparticles production are used as alternatives to physicochemical methods. Such methods could produce MgO-NPs in an effective, safe, and environmentally friendly manner; in addition, the produced particles are highly stable (Fouda et al., 2021d). Besides that, magnesium is one of the important elements required for plant growth and photosynthesis process. Moreover, the FDA has approved MgO-NPs as a safe and effective antibacterial alternative (Kumar et al., 2020).

Recently, algal biomass, whether dead (dried) or alive, has attracted increasing attention as an environmentally friendly cell factory for the rapid and effective green synthesis of nanoparticles (Uzair et al., 2020; El-Belely et al., 2021). Algae have many unique properties such as rapid growth rate, huge biomass productivity, and capacity for accumulation and reduction of mineral ions. Microalgae and macroalgae can grow without the aid of fertilizers or chemicals. Unlike many biomasses, algae are harvested several times a year (Jacob et al., 2021). It is convenient to utilize algae as nano-factory in a simple water medium at ambient pressure and temperature at natural pH (Mukherjee et al., 2021). Byproducts of algal metabolism can produce metallic, bimetallic, or metal oxide nanoparticles by reducing, capping, and stabilizing metal precursors (Chaudhary et al., 2020).

*Cystoseira crinita* Duby is a marine macroalga that belongs to the class of Phaeophyceae (Brown Algae). Brown macroalgae are characterized by powerful biomass that produces various biologically active substances that can act as an effective reducing and stabilizing agent during nanoparticles biosynthesis. Thus, macroalgae-mediated biosynthesis of nanoparticles has become an attractive approach due to the abundance of these biologically active marine resources (Kim and Chojnacka, 2015). Recently, González-Ballesteros and coauthors reported the biogenic synthesis of gold nanoparticles by the marine macroalgae *Cystoseira baccata* (González-Ballesteros et al., 2017). The extract of *C. crinita* has been reported to have antibacterial, antioxidant, antiviral and cytotoxic, anti-inflammatory, and antiproliferative activities (Bruno De Sousa et al., 2017). However, to the best of our knowledge, there is no report on the green synthesis of MgO-NPs by *C. crinita.*

The biosynthesize of MgO-NPs was previously reported using fungal strains that manifested potent antimicrobial properties against the selected pathogens, *Candida albicans*, *Bacillus subtilis*, *Staphylococcus aureus*, *Escherichia coli,* and *Pseudomonas aeruginosa*, in addition to their catalytic activity for reducing the physicochemical properties and chromium ion content of tanning effluents, along with their adult and larvicidal repellence capacity against *Culex pipiens* (Hassan et al., 2021; Saied et al., 2021). Furthermore, the Phyto-synthesized MgO-NPs displayed remarkable antimicrobial properties and their toxicological profile revealed powerful toxicity against the breast cancer cell lines (MCF-7), as well as the boosted photocatalytic degradation of MgO-NPs for methylene blue (Amina et al., 2020).

Considering the phyco-nanotechnology, the current study was designed for single-step biosynthesis of magnesium oxide nanoparticles using the aqueous extract of the marine brown macroalgae *Cystoseira crinita*. The characterization of the bio-fabricated nanoparticles was fulfilled by UV-Vis spectroscopy, XPS, TEM, XRD, SEM-EDX, and FT-IR analyses. The biomedical performance of the MgO-NPs comprising antimicrobial, *in-vitro* cytotoxicity, larvicidal, and repellency efficiency was evaluated.

## Materials and Methods

### Materials Used

All chemicals used are analytical grades obtained from Sigma Aldrich, Cairo, Egypt. Magnesium nitrate hexahydrate [Mg(NO_3_)_2_.6H_2_O] was used as a precursor for MgO-NPs synthesis. The antimicrobial activity was conducted using Muller Hinton agar media (ready-prepared-Oxoid), whereas the cell lines used to investigate the *In-vitro* cytotoxicity were obtained from the Holding Company for Biological Products and Vaccines (VACSERA), Dokki, Giza, Egypt. All the reactions in the current study were conducted using distilled water (dis. H_2_O).

### Macroalgae Biomass Collection

Biomass of the brown algae *C. crinita* was collected from the Western Red Sea coast of Egypt in Hurghada City (27° 17′ 02.5″ N, 33° 46′ 21.0″ E).

### Macroalgae Aqueous Extract Preparation and Biosynthesis of MgO-NPs

**FIGURE 1:**
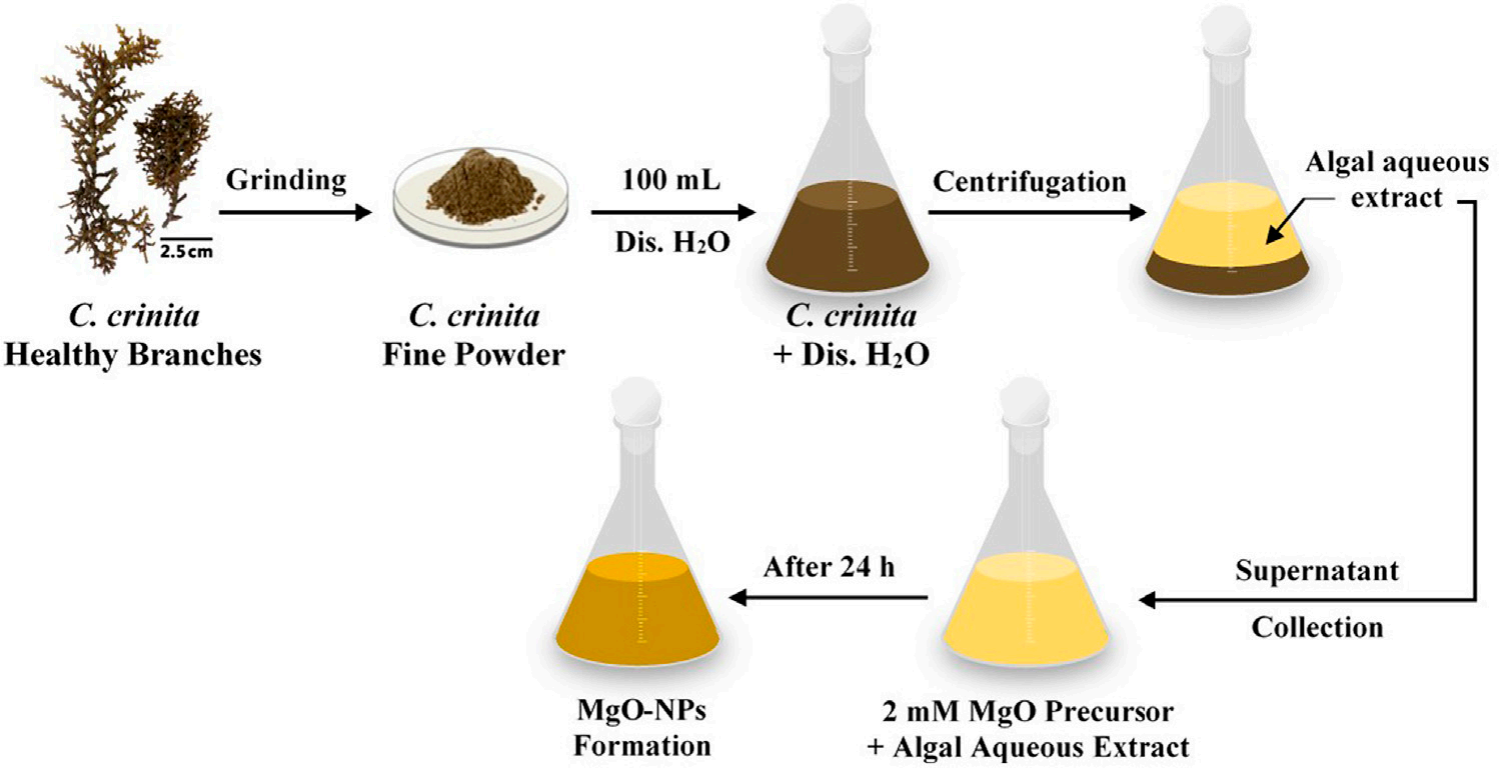
A flowchart shows the biosynthesis of MgO-NPs by the brown algae *Cystoseira crinita*.

The collected biomass of *C. crinita* was washed several times with tap water to remove seawater salts followed by washing with double-distilled water to remove any attached debris and sediments. The epiphytes were removed manually. The washed samples were oven-dried at 70°C for 24 h, followed by grinding into a fine powder using an electric blender. Approximately 10 g of *C. crinita* powder was mixed with 100 ml dis H_2_O, shaken well, and heated at 60°C using a magnetic stirrer for 120 min. After that, the mixture was centrifuged for 10 min at 1500 *rpm*, and the supernatant was collected and used as reducing and stabilizing for MgO-NPs as follows: 51.3 mg of metal oxide precursor [Mg (NO_3_)_2_.6H_2_O] was dissolved in 10 ml dis. H_2_O and added to 90 ml of obtained algal aqueous extract to get a final concentration of 2 mM (Figure 1). After 24 h of incubation, the change of color from pale yellow to yellowish-brown indicates the formation of MgO-NPs (Pugazhendhi et al., 2019). Finally, the resultant NPs were calcinated at 400°C for 4 h.

### Characterization of Synthesized MgO-NPs

The maximum surface plasmon resonance (SPR) of biosynthesized MgO-NPs was detected through monitoring the absorbance spectra using a UV-Vis spectrophotometer (Jenway 6305, Staffordshire, United Kingdom) in the range of 200–800 nm. The role of functional groups present in the aqueous extract of *C. crinita* in the reduction and stabilizing processes was investigated by Fourier transform infrared (FT-IR) spectroscopy (Agilent system Cary 660 FT-IR model). The NPs sample was mixed with KBr and pressured to form a disk that was scanned in the range of 400–4000 cm^−1^ (Badawy et al., 2021). The shapes of biosynthesized MgO-NPs and their sizes are investigated using Transmission Electron Microscopy (TEM) (JEOL 1010, Japan, acceleration voltage of 120 kV, X40000). A drop of MgO-NPs colloidal solution was added to the TEM grid and the solution excess was removed by contacting the gride to blotting paper. The loaded gride was undergone vacuum desiccation for 24 h before being placed on the TEM holder (Soliman et al., 2021). The elemental contents in the MgO-NPs sample were analyzed using Scanning Electron Microscopy linked with energy-dispersive X-ray (SEM-EDX) (JEOL, JSM-6360LA, Japan) (Shaheen and Fouda, 2018).

The X-ray diffraction (XRD) analysis was conducted by X’Pert PRO at a 2θ degree of 0°–80° (Philips, Eindhoven, Netherlands). The operation conditions were voltage at 40 kV, current at 30 mA, and the x-ray source was Cu Ka radiation. Based on XRD analysis, the Debye–Scherrer equation was used to measure the particle size (Gu et al., 2018) as follows:

D=0.9×0.154/β Cosθ (1)

where D is average particle size, 0.9 is the Scherrer’s constant, 0.154 is the wavelength (nm) of X-ray radiation, β is the half of maximum intensity, and θ is the Bragg’s angle.

The X-ray photoelectron spectroscopy (XPS) analysis was accomplished by ESCALAB 250XI^+^ instrument (Thermo Fischer Scientific, Inc., Waltham, MA, USA) connected with monochromatic X-ray Al Kα radiation (1,486.6 eV). The analysis was conducted under the following conditions: the size of the spot was 500 μm, the samples were prepared under a pressure adjusted at 10^–8^ mbar, the energy was calibrated with Ag3d_5/2_ signal (∆BE: 0.45 eV) and C 1s signal (∆BE: 0.82 eV), and the full and narrow-spectrum pass energies were 50 and 20 eV, respectively (Hamza et al., 2021a).

### Biological Activities

#### Antimicrobial Activity

The activity of phyco-synthesized MgO-NPs against pathogenic Gram-positive bacteria (*Staphylococcus aureus* ATCC6538, *Bacillus subtilis* ATCC6633), Gram-negative bacteria (*Pseudomonas aeruginosa* ATCC9022, *Escherichia coli* ATCC8739), and unicellular fungi (*Candida albicans* ATCC10231) was investigated by the agar well diffusion method. Briefly, the bacterial strains and *C. albicans* were inoculated on nutrient broth media and yeast extract peptone dextrose (YEPD), respectively, and incubated at 35.0 ± 2°C for 24 h (Fouda et al., 2021b). At the end of the incubation period, 50 µL of each microbial strain (adjusted O.D. at 1.0) were seeded onto Muller Hinton agar media, shaken well, and poured into Petri dishes under sterilized conditions. After the solidification, wells (0.7 mm) were made in seeded plates before being filled with 100 µL of stock MgO-NPs solution (200 μg mL^−1^). The loaded Muller Hinton plates are kept in the refrigerator for one hour before being incubated at 35.0 ± 2°C for 24 h (Fouda et al., 2021a). At the end of the incubation period, the results were recorded as a diameter of the zone of inhibition (mm) that appeared around each well. The activity of different MgO-NPs concentrations (100, 50, 25, and 12.5 μg mL^−1^) was also investigated in the same manner to detect the minimum inhibitory concentration (MIC) value for each test organism. The experiment was achieved in triplicates.

#### *In-vitro* Cytotoxicity

Cell culture used: Two cell lines represented by Vero cells (kidney of African green monkey) as normal cells and Caco-2 cells (colon carcinoma cell) as cancerous cells.

MTT assay method. The *in-vitro* cytotoxic efficacy of phyco-synthesized MgO-NPs was studied by the cell viability MTT [3-(4,5-dimethylthiazol-2-yl)-2,5-diphenyl tetrazolium bromide] assay method. Briefly, the selected cell lines were grown separately in a 96-well microtiter plate at a concentration of 1×10^5^ cell/well. The inoculated plates were treated by the double-fold MgO-NPs concentration (500, 250, 125, 62.5, 31.25, 15.6, and 7.8 μg mL^−1^) and incubated at 37°C for 48 h. At the end of the incubation period, MTT reagent (5 mg mL^−1^ in phosphate buffer) was added to each well and incubated under 5% CO_2_ at 37 °C for 1–5 h. After that, the purple formazan crystal (MTT metabolic product) was formed which was dissolved by the addition of 10% DMSO. The plates were subjected to agitation for 30 min in dark conditions and followed by the measure of the formed color intensity at 560 nm by an enzyme-linked immunosorbent assay (ELISA) plate reader (Lashin et al., 2021). The cell viability percentages were measured according to the following equation:

$$\mathrm{Cell}\;\mathrm{viability}\;\mathrm{percentages}\left(\%\right)=\frac{\mathrm{Absorbance}\;\mathrm{of}\;\mathrm{treated}\;\mathrm{sample}}{\mathrm{Absorbance}\;\mathrm{of}\;\mathrm{control}}\mathrm{\times 100}$$ (2)

#### Mosquitocidal Bioassay

##### Larvae Rearing

The laboratory-bred colony of *Musca domistica* (housefly) was obtained from animal houses, Flies Research Laboratory, Faculty of Science, Al-Azhar University, Cairo, Egypt. Larval and pupae instars were then reared in a plastic cup containing dried Brewer’s beans, yeast extract, and milk powder in standard conditions of 28.0 ± 2.0°C (Hogsette, 1992).

##### Larvicidal and Pupicidal Bioassay

Larval bioassay was estimated using the dipping method according to Sinthusiri and Soonwera (2010). Five concentrations of MgO-NPs were prepared (2, 4, 6, 8, and 10 μg mL^−1^), 25 third instar larvae were dipped in 10 ml of each concentration of the nano-scaled colloidal MgO for 30 s and then transferred to filter paper (in a plastic beaker). Three replicates were prepared for each concentration, while control larvae were dipped in distilled water for 30 s. Larval mortality percentages were recorded after 24 h of treatment according to the following equation (Fouda et al., 2020):

Mortality percentages(%)=A-B B x100 (3)

where A is the mortality percentages in treatment and B is the mortality percentages in control.

##### Repellent/Attraction Assay

Twenty newly emerged mixed-sex adults were housed in a cage (18 × 24 × 18 inches) containing two conical flasks. One of the two flasks contained 1% MgO-NPs (10 μg mL^−1^) in 5 ml of milk, while the other contained 5 ml of milk to be used as a control. A funnel (4 inches in diameter) was inserted into each flask to prevent the escape of flies. The total number of flies trapped in these flasks was counted after 24 h. The results were expressed in terms of the attraction/repulsion ratio. The repellence percentage (R, %) was calculated by the following formula (Campbell, 1983):

$$\mathrm{Repellence}\;\mathrm{percentages}\left(\%\right)=\frac{\mathrm{C-T}}{C}\mathrm{x100}$$ (4)

where C, is the number of flies restricted in the control flask and T, is the number of flies restricted in the treated flask. This experiment was performed with five repetitions.

### Statistical Analysis

All the results presented are the means of three independent replicates. Data were subjected to statistical analysis by a statistical package SPSS v17. The mean difference comparison between the treatments was analyzed by *t*-test or the analysis of variance (ANOVA) and subsequently by Tukey HSD test at *p <* 0.05.

## Results and Discussion

### UV-Vis Spectroscopy Analysis

**FIGURE 2:**
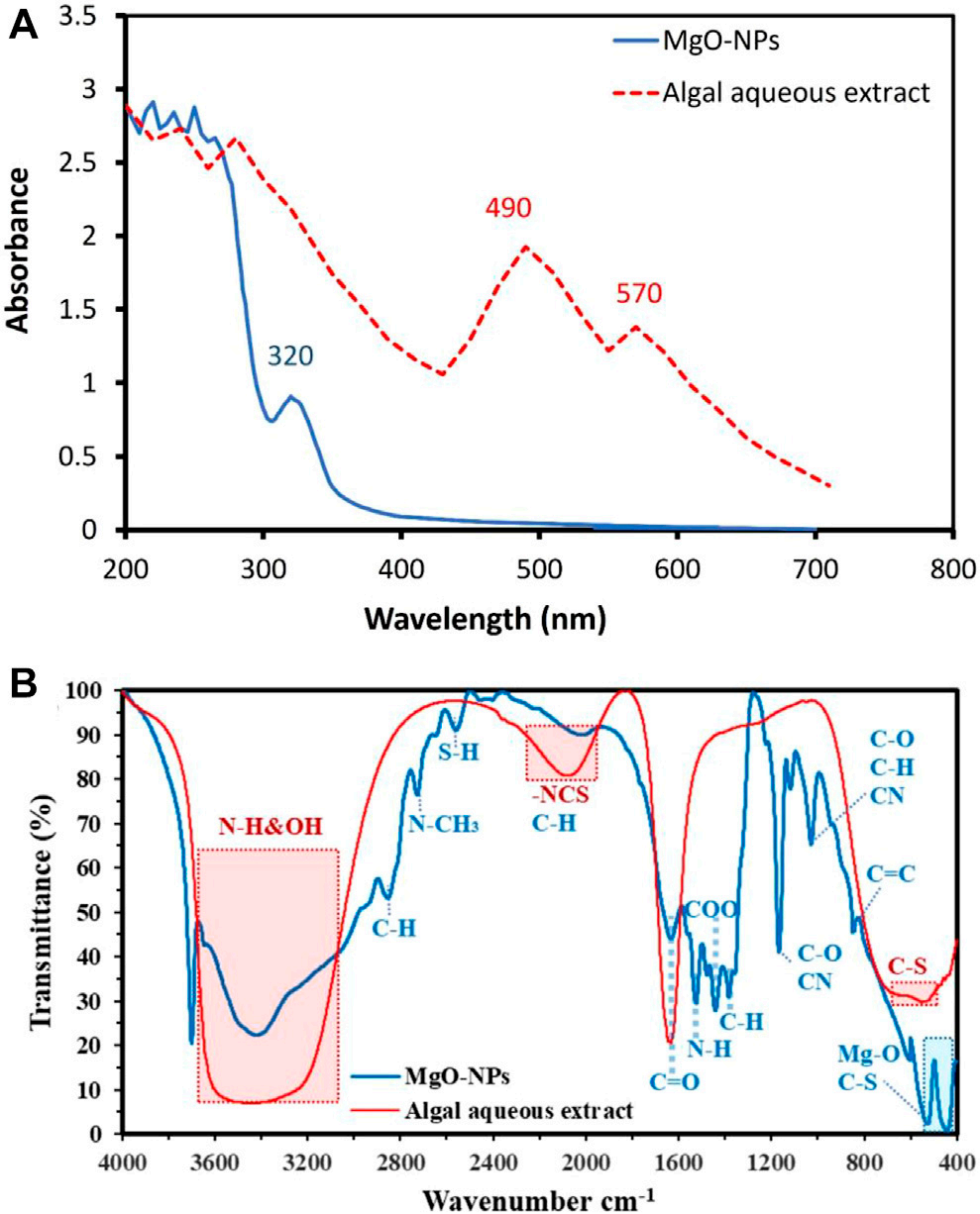
UV-vis spectroscopy **(A)** and FT-IR **(B)** for algal aqueous extract and MgO-NPs synthesized by brown algae *C. crinita.*

The first monitor for phyco-synthesis of MgO-NPs was the color change from pale yellow to yellowish-brown due to the mixing of aqueous extract of *C. crinita* with Mg(NO_3_)_2_.6H_2_O. This change was investigated by UV-vis spectroscopy to detect the maximum surface plasmon resonance (SPR), which mainly depends on the size, distribution, and shape of phyco-synthesized MgO-NPs in the colloidal solution (Fedlheim and Foss, 2001). In this reaction, the UV radiations react with the metals in the tested solution that support the transition of electrons from low to a higher energy state, then the SPR is obtained which gives a prediction about the shape and size of NPs in the range of (2–100 nm) (Poinern, 2014). In this study, the maximum SPR was appeared at 320 nm, whereas algal aqueous extract has two maximum peaks at 490 and 570 nm (Figure 2A). The shifting in maximum peak confirmed the successful formation of MgO-NPs by algal metabolites present in the aqueous extract that act as reducing and capping/stabilizing agents. Compatible with the current study, the aqueous extract of brown algae *Sargasssum wightii* was changed to yellowish-brown after being mixed with Mg(NO_3_)_2_ as an indication of MgO-NPs formation, and the maximum SPR was appeared at 322 nm (Pugazhendhi et al., 2019). The presence of adsorption peaks at wavelength 200–250 nm refers to the existence of different bioactive compounds in algal aqueous extract such as alginates and polyphenols that play a critical role in the reduction of Mg(NO_3_)_2_.6H_2_O to form MgO-NPs (Pugazhendhi et al., 2019).

The synthesis of NPs in our study is extracellular mode that is more convenient as the produced NPs are easily purified. Several compounds aid as reducing agents. As previously reported, the dominant biomolecules in brown algae are sterols such as cholesterols, fucosterols, sulfated polysaccharides, and various functional groups including glucuroronic acid, muramic acid, alginic acid, and vinyl derivatives that could act as reducing as well as capping agents for the synthesis of NPs (Chaudhary et al., 2020). For synthesis conditions, the pH of reaction medium is one of the important used experimental parameters in our synthetic method (Fouda et al., 2018). Here, the conducted alkaline condition used for NPs synthesis, as compared with neutral condition, enhances the reducing power of functional groups and prevents the NPs agglomeration (Parial et al., 2012). Besides that, it helps in the NPs capping and stabilization by interacting with the amine groups of surface-bound proteins and their residual amino acids (Namvar et al., 2014). Further experiments for the effect of different pH values, temperature, time, static conditions, substrate concentration, and stirring to find the optimal reaction conditions and the best physical characteristics of NPs should be investigated for the validation of all experimental conditions.

### Fourier Transform Infrared Spectroscopy

The bioactive compounds present in the aqueous extract of brown algae *C. crinita* and their role in the reduction, capping, and stabilizing of MgO-NPs were identified by FT-IR analysis (Figure 2B). FT-IR of the algal aqueous extract confirms the presence of C=O from polysaccharide moieties at 1627 cm^−1^, whereas the broadband at 2075 cm^−1^ is related to -NCS stretching of fucoidan from biomass that explains the sulfone stretching peak (Hamza et al., 2020a; Hamza et al., 2020b). The strong broadness of bands at 3340 related to OH and NH stretching vibration. The appearance of absorption peaks in the range of 450–650 cm^−1^ refers to the successful formation of Mg―O (Ramanujam and Sundrarajan, 2014; Pugazhendhi et al., 2019; Fouda et al., 2021c); this peak overlapped with the C-S band from the biomasses (Hamza et al., 2021b). The weak absorption peak at 840 cm^−1^ corresponded to bending C═C of alkene. The peak at 1027 cm^−1^ related to stretching C―O or bending C―H or stretching CN of primary amines (Coates, 2006; Hamza et al., 2019), whereas the peak at 1166 cm^−1^ corresponded to stretching C―O of the alcohol and overlapped with stretching C―N of tertiary amines (Coates, 2006). The absorption peak at 1395 cm^−1^ is related to the vibration of C―H bending (Ogunyemi et al., 2019), whereas the peak at 1438 cm^−1^ is corresponded to stretching C═O of carboxylic salts and adsorption of CO_2_ and CO_3_^2–^ on the MgO-NPs surface (Coates, 2006; Hamza et al., 2021b). The absorption peak at 1628 cm^−1^ is related to water adsorption in the sample (Moorthy et al., 2015), whereas the broad peak at 2000 cm^−1^ is signified to C―H bending of aromatic compounds overlapped with -NCS of biomass (Hamza et al., 2020a). The weak absorption peak at 2557 cm^−1^ is referred to stretching of the S―H group of thiol-containing compounds, whereas the peak at 2723 corresponds to N―CH_3_ methylamine and stretching C―H bond (these data are matched with XPS analysis) (Coates, 2006). The small absorption peak at 2850 cm^−1^ signifies to stretching vibration bond of C―H in CH_2_ groups’ existence in the phyco-chemical compounds (Essien et al., 2020). Finally, the broad and strong peaks that appeared in the range of 3400 cm^−1^ to 3700 cm^−1^ corresponded to the N―H and O―H groups of different amino acids present in the aqueous extract of brown algae (Moorthy et al., 2015; Pugazhendhi et al., 2019). Jena and coworkers reported that the most common functional groups present in the algal aqueous extract and responsible for NPs fabrications are –NH_2_–, –C═O–, and –SH– groups (Jena et al., 2014). According to the FT-IR analysis, the role of bioactive compounds such as amino acids, polysaccharides, primary and tertiary amines, and others that are present in the aqueous extract of brown algae has been confirmed to act as reducing, capping, and stabilizing of MgO-NPs.

### Transmission Electron Microscopy

**FIGURE 3:**
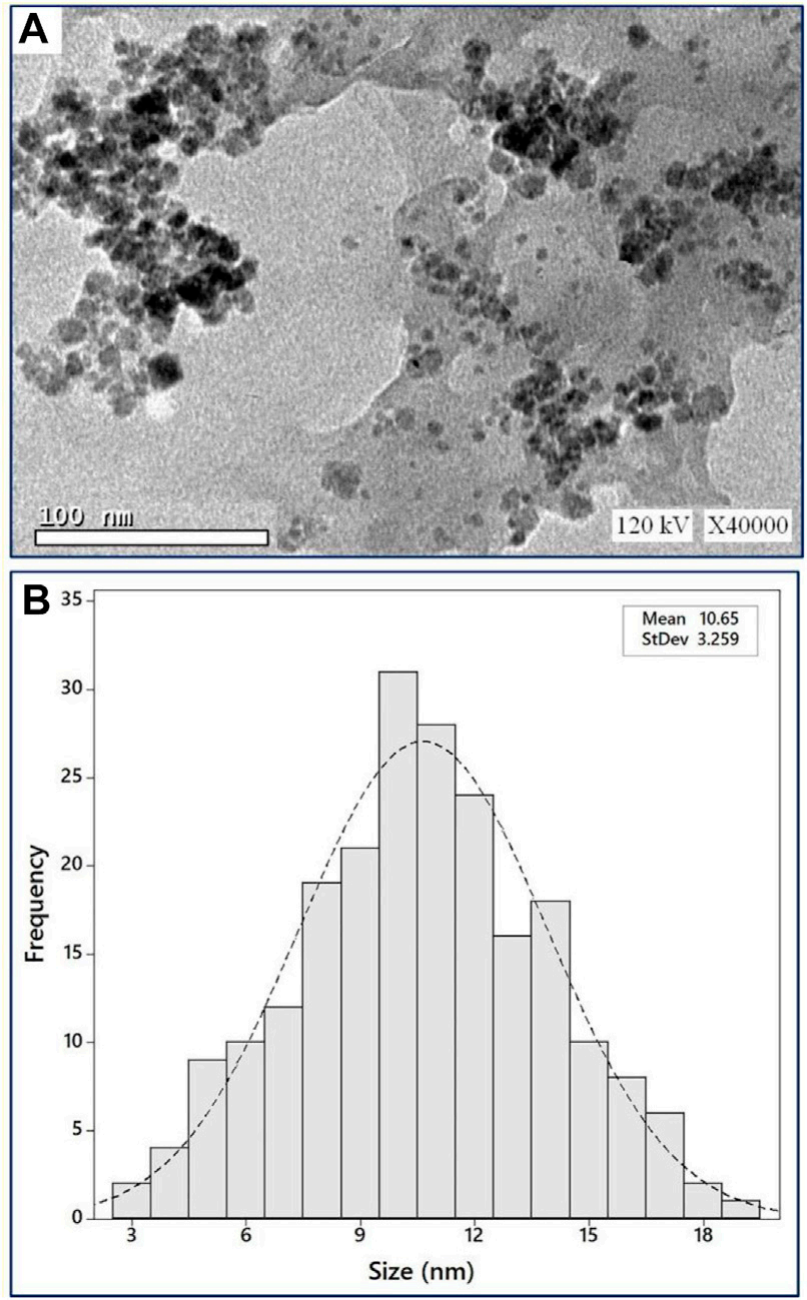
Characterization of green synthesized MgO-NPs by brown algae *C. crinita*. **(A)** TEM image showed spherical shape, **(B)** size distribution of particles based on TEM image.

The activity of NPs is usually correlated with different characters including shape, size, and distribution (Salem and Fouda, 2021). Therefore, it is important to detect the size and shape of NPs. As shown from the TEM analysis in Figure 3A, the bioactive compounds present in the aqueous extract of macroalgae *C. crinita* have the efficiency to fabricate spherical and well-dispersed MgO-NPs with a size range of 3.0–18.0 nm with an average size diameter of 10.65 ± 3.3 nm (Figure 3B). As mentioned in the previous studies, the activity of NPs is increased by decreasing their size. For example, the activity of MgO-NPs to inhibit the growth of pathogenic Gram-positive *Bacillus subtilis* was varied according to the sizes used. The inhibition percentages were 75.7%, 94.5%, and 96.1% for size 2145.9, 47.3, and 35.9 nm, respectively (Huang et al., 2005). Moreover, spherical MgO-NPs synthesized through harnessing metabolites of *Penicillium chrysogenum* exhibited a size range of 7–40 nm and showed inhibition activity against pathogenic Gram-positive bacteria, Gram-negative bacteria, and unicellular fungi as well against larvae and pupa of malarial vector *Anopheles stephensi* (Fouda et al., 2021a). The obtained size (3.0–18.0 nm) of MgO-NPs in the current study is expected to have high activity for various biomedical and biotechnological applications.

### Energy-Dispersive X-Ray Analysis

**FIGURE 4:**
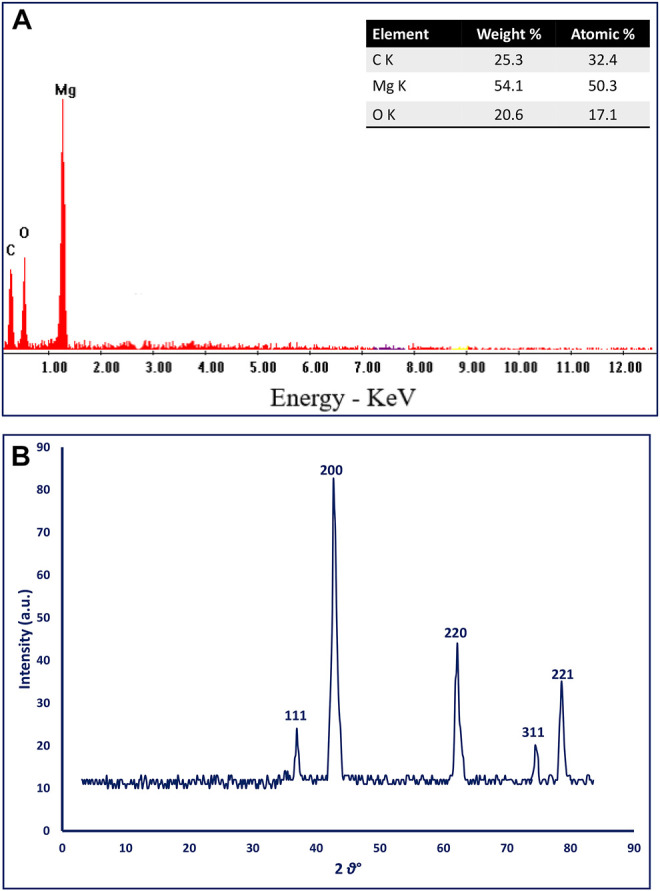
**(A)** The EDX chart, and **(B)** XRD analysis of algal-mediated green synthesized MgO-NPs.

The EDX analysis is used to detect the elemental composition of the biosynthesized MgO-NPs. The EDX chart showed that the phyco-synthesized MgO-NPs are highly pure; it contains Mg and O ions which indicates the successful formation of MgO through harnessing metabolites present in the aqueous extract of brown algae *C. crinita* (Figure 4A)*.* Also, the existence peak of Mg and O at bending energy between 0.5 and 1.5 KeV confirms the successful formation of MgO (Fouda et al., 2021e). The quantitative analysis revealed that the weight percentages of Mg and O ion in the sample were 54.1 and 20.6%, respectively, whereas the atomic percentages were 50.3% and 17.1%, respectively (Figure 4A). Similarly, the EDX profile of MgO-NPs fabricated by the *Pterocarpus marsupium* aqueous extract showed two peaks at 0.5 and 1.5 KeV for O and Mg ions with weight percentages of 69.3 and 30.6%, respectively (Ammulu et al., 2021). The presence of C indicates the bounding of algal metabolites to the MgO-NPs surface. Compatible with the current study, the presence of carbon in the EDX chart of MgO-NPs was attributed to the attached biomolecules secreted by brown algae *Sargasssum wightii* to the surface of MgO (Pugazhendhi et al., 2019).

### X-Ray Diffraction Analysis

The crystallinity of phyco-synthesized MgO-NPs was investigated using the XRD pattern. The data represented in Figure 4B showed five intense peaks at 2θ values of 36.9°, 42.6°, 62.2°, 74.7°, and 78.8° which corresponded to (111), (200), (220), (311), and (222), respectively. The obtained peaks confirmed that the phyco-synthesized MgO-NPs were a crystallographic and face-centered cubic structure (FCC) as compared with JCPDS file no.39–7746 (Pugazhendhi et al., 2019). The size of biosynthesized MgO-NPs can be calculated according to the width of the sharp XRD peak (200) located at a 2θ value of 42.7° by Debye Scherrer’s equation. Data showed that the mean crystal size of phyco-synthesized MgO-NPs was 21 nm based on XRD analysis. Similar XRD spectra were recorded for green synthesized MgO-NPs by different biological entities (Sharma et al., 2020; Ammulu et al., 2021; Hassan et al., 2021).

### X-Ray Photoelectron Spectroscopy Analysis

**FIGURE 5:**
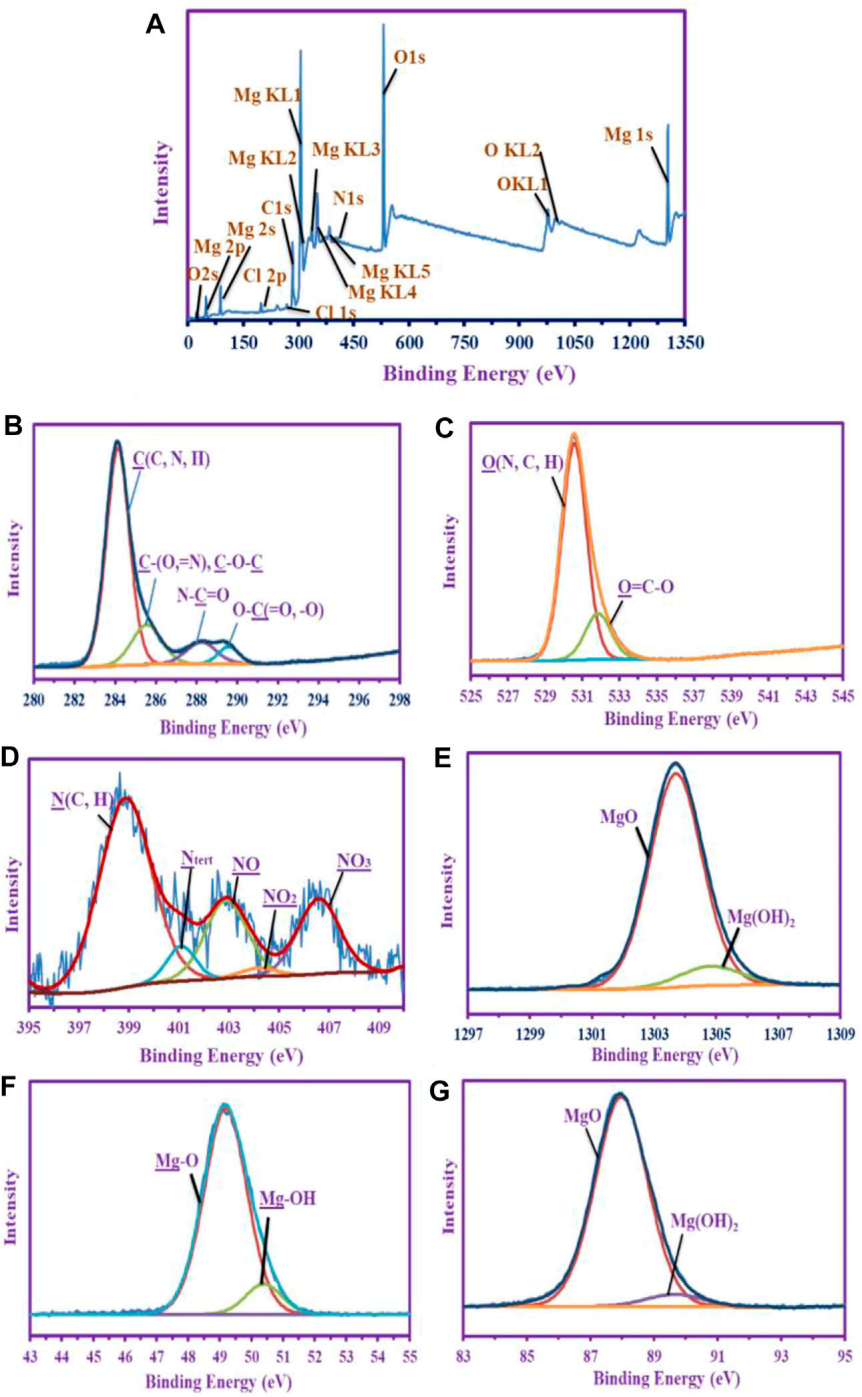
XPS analysis of MgO-NPs synthesized by harnessing metabolites of brown algae *C. crinita*. **(A)** Total analysis an overview; **(B)** denotes the C 1s; **(C)** denotes the O 1s; **(D)** denotes the N 1s; **(E)** denotes the Mg 1s; **(F)** denotes the Mg 2p; and **(G)** denotes the Mg 2s.

The XPS analysis of MgO-NPs was analyzed and characterized by several identified peaks. Figure 5A depicts the survey analysis spectra of the sample, which has several peaks of the constituents; this appears in C 1s, N 1s, Cl (1s, 2p), O (1s, KL1, 2s, and KL2), whereas the Mg was detected at different bending energy (BE) as 1s, 2s, 2p, and KL1-5 verifying a majority product of this element over the other elements.

Different profiles of deconvolutions and signals were shown in comparing the chemical constituents. The deconvoluted and assignment peaks of the most abundant groups or species are recorded. The C 1*s* shows five splitting internal peaks, which are assigned as C (C, N, H) at 284.09 eV, C(=N, O) or C-O-C at 285.51 eV, N-C=O (amide) at 288.18 eV, and O-C=O and O-C-O at 289.53 eV, respectively (Figure 5B) (Jurado-López et al., 2017; Hamza et al., 2019; He et al., 2021). Two internal splitting peaks were shown for the O 1s peak (Figure 5C) which was assigned for O (N, C, H) at 530.52 eV, and the other peak for O-C=O at 531.8 eV (Jurado-López et al., 2017; Hamza et al., 2021b; Wei et al., 2021). This proves the chemical composition of the hydrocarbon (i.e., carbohydrate moiety) produced by the brown algae is compatible with the EDX chart.

The N 1s was splitting into five internal peaks assigned at 398.82 eV for N (C, H), and 401.04 eV for N_tert_, this is for polysaccharides moieties (Lu et al., 2019; He et al., 2021), whereas the medium of the sorption and the dissolved salt source was appeared by three peaks 402.84, 404.15, and 406.54 eV for NO, NO_2_, and NO_3_, respectively (Figure 5D) (Bartis et al., 2015).

As shown in Figures 5E–G the Mg is predominately an oxide over the hydroxide species; this was shown in the Mg1s which split into two peaks at 1303.65 eV with (at%) 88.83, and the other peak at 1304.77 eV for Mg(OH)_2_ with 11.7% (at%) (Yao et al., 2013). Mg2p shows the same deduction as in the Mg1s, the majority for the MgO at 49.14 eV, (at%) 88.77 over Mg-OH 50.34 eV (at% 11.23%) (Dang et al., 2017; Le Febvrier et al., 2017), whereas the Mg2s deconvoluted twice at 87.93 eV with 90.76% (at%) for MgO and at 89.6 eV with 9.24% (at%) for Mg(OH)_2_ (Dang et al., 2017). This emphasizes the predominantly abundant MgO species over Mg(OH)_2_ species and the majority of Mg in the yield.

### Antimicrobial Activity of MgO-NPs

**FIGURE 6:**
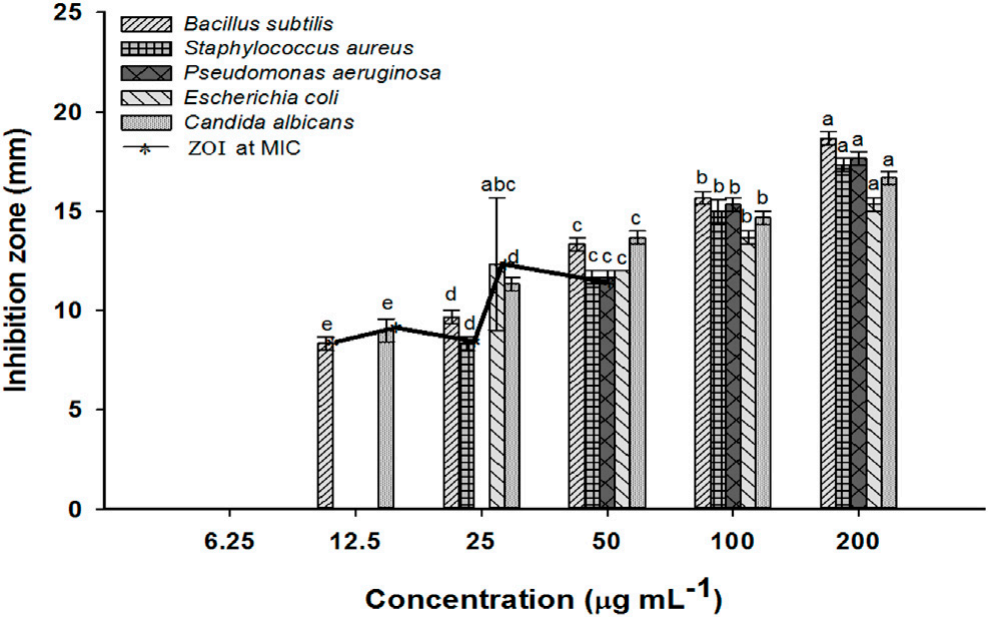
The antimicrobial activity of MgO-NPs at different concentrations against Gram-positive and Gram-negative bacteria, and unicellular fungi and inhibition zones at MIC. Different letters at the same concentration indicate the significant vales (*p ≤* 0.05).

The agar well diffusion assay was used to evaluate the microbicidal activities of the phyco-synthesized MgO-NPs. Measurement of inhibition zones developed around agar wells demonstrated the dose-dependent activity of MgO-NPs against selected clinical pathogens (*Bacillus subtilis*, *Staphylococcus aureus*, *Pseudomonas aeruginosa*, *Escherichia coli*, and the unicellular fungi (*Candida albicans*) as recently reported (Hassan et al., 2021). The results proved the divergent activity of the nanostructured MgO against all tested pathogens. The MgO-NPs at 50 μg mL^−1^ exhibited a wide spectrum activity against all tested microbes. *Bacillus subtilis* was the most sensitive strain recording 13.3 ± 0.5 mm zone of inhibition (ZOI). Doubling the concentration of MgO-NPs (100 μg mL^−1^) improved the antimicrobial activity, and *B. subtilis* retained the biggest ZOI (15.6 ± 0.5 mm), whereas *E. coli* had the smallest ZOI (13.6 ± 0.5 mm). The MgO-NPs concentration at 200 μg mL^−1^ showed the maximum effectiveness against all microbes, *B. subtilis* recorded the largest ZOI (18.6 ± 0.5 mm), whereas it was 15.3 ± 0.5 mm for *E. coli*. Moreover, *S. aureus*, *P. aeuroginosa*, and *C. albicans* recorded 17.3 ± 0.5, 17.6 ± 0.5, and 16.6 ± 0.5 mm ZOIs, respectively **(** Figure 6).

MgO-NPs synthesized by brown algae *Sargassum wighitii* showed antifungal and broad-spectrum antibacterial potential against *Proteus mirabilis*, *Staphylococcus aureus*, *Serratia marcescens*, *Escherichia coli*, *Salmonella typhimurium*, and *Pseudomonas aeruginosa* (Pugazhendhi et al., 2019). Similarly, Deepak and coworkers demonstrated the facile fabrication of silver-NPs by the brown seaweed *Sargassum wightii*, and they reported bactericidal efficacy against *B. subtilis*, *B. cereus*, *P. aeruginosa*, *Salmonella typhimurium*, *Enterococcus faecalis*, and *Shigella flexneri* (Deepak et al., 2018).

MgO-NPs are metal-based, light nanoparticles with antimicrobial potential; it is totally resorbed and metabolized in the body. The minimal fungicidal and bactericidal inhibitory concentrations of MgO-NPs against prevailing infectious yeasts and bacteria must be determined to be applied clinically (Nguyen et al., 2018). Herein, MgO-NPs inhibited the growth of all the tested microbes. However, MIC was divergent for the two Gram-positive bacteria, specifically 12.5 μg mL^−1^ was the MIC for *B. subtilis* and 50 μg mL^−1^ for *S. aureus*. In contrast, the concentration of MgO-NPs increased to 25 and 50 μg mL^−1^ for realizing the MIC for the Gram-negative bacteria, *P. aeruginosa,* and *E. coli*, respectively. The antifungal properties of MgO-NPs were confirmed against *C. albicans*, registering the MIC value 12.5 μg mL^−1^.

**FIGURE 7:**
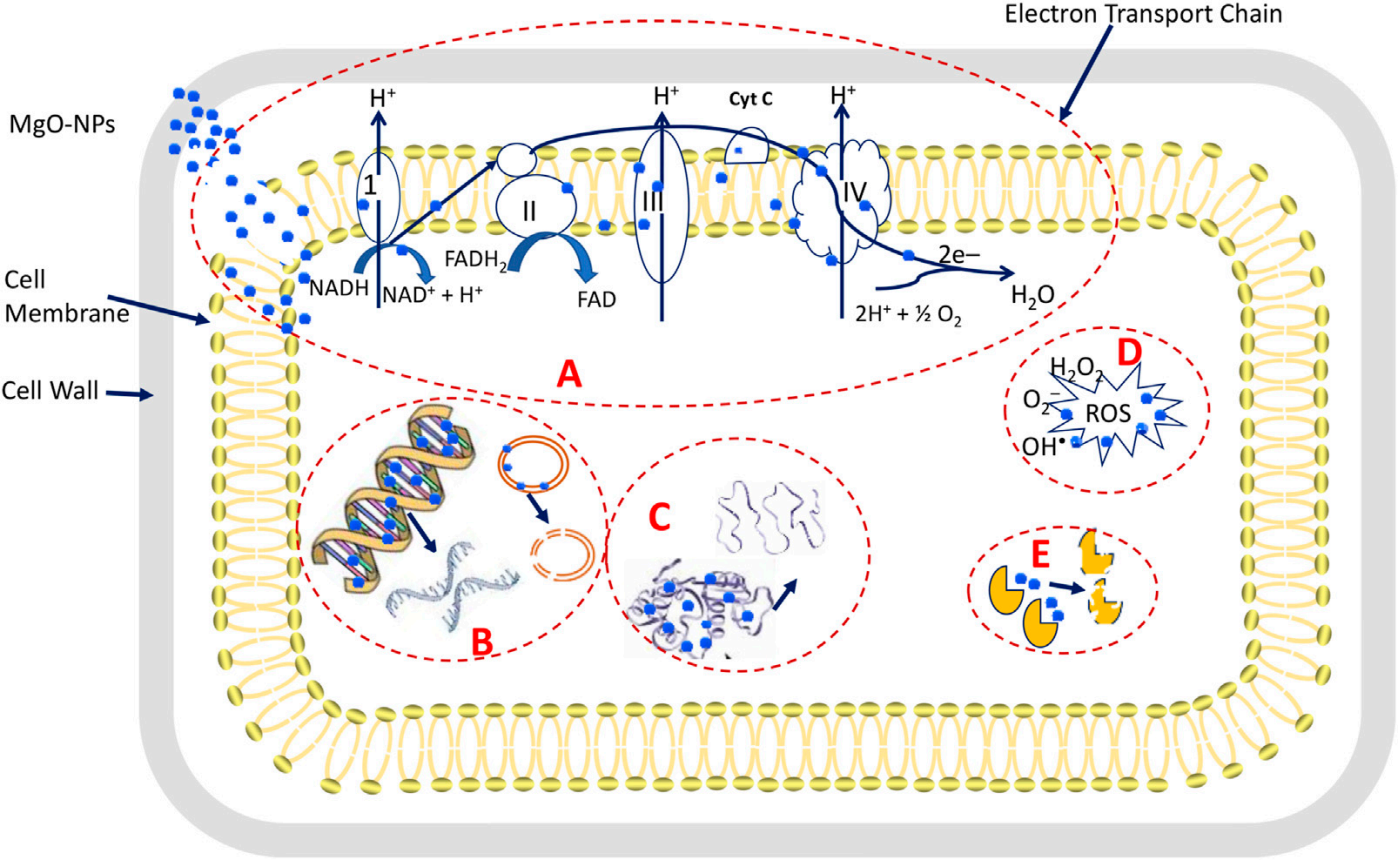
Proposed antimicrobial mechanisms of MgO-NPs. **(A)** is the adhesion of MgO-NPs with the cell wall and electron transport chains ultimately disrupting the selective permeability function, **(B)** is the entrance of MgO-NPs into the cell and reacting with DNA and plasmids lead to genotoxicity, **(C)** is reacting of MgO-NPs with proteins ultimately to denaturation, **(D)** is the production of toxic substances that enhance the ROS that leads to macromolecules disruption. And **(E)** is blocking and changing the active sites in enzymes because of reacting with MgO-NPs.

The antibacterial properties of MgO-NPs are attributed to various mechanisms as proposed by several authors as shown in Figure 7. These mechanisms include the enhancing reactive oxygen species (ROS) production, reaction of MgO-NPs with the bacterial cell wall and interfering with electron transport chains, the interaction between Mg^2+^ and active macromolecules inside the bacterial cells, and the production of alkaline conditions due to the release of Mg^2+^ inside the bacterial cells (Wong et al., 2020). The interaction between the bacterial cell wall and MgO-NPs might lead to the deformation of a cell wall structure and disrupt the electron transport chains, ultimately blocking the selective permeability function (Karthik et al., 2019). Also, this interaction led to the formation of toxic substances such as ^•^OH, H_2_O_2_, and^–^O_2_ that irreversibly destroy the cell wall and its important components such as phospholipids. It might also interfere with protein and nucleic acids, which necessitates cell death (He et al., 2016). In addition, once MgO-NPs enter the cell, it presents their alkaline effects with the release of Mg^2+^ (Karthik et al., 2019; Abinaya et al., 2021). Likewise, the efficient toxicity of MgO-NPs against diverse multidrug-resistant clinical pathogens makes it a good candidate for alternative medicine (He et al., 2016).

### *In-Vitro* Cytotoxicity of MgO-NPs Against Normal and Cancer Cells

Various nanoparticles showed anticancer properties and can be used in cancer therapy or as targeted delivery systems for anticancer drugs (Chaudhary et al., 2020). The safe use of NPs as an antiproliferative agent against carcinoma requires evaluation of their side effects against normal cell lines and protein structure (Behzadi et al., 2018). Consequently, an MTT assay was used to evaluate the cytotoxicity of the phyco-synthesized MgO-NPs against the human colon cancer cell lines (Caco-2) and the monkey’s healthy cell lines (Vero). The MTT assay colorimetrically estimates the viability and proliferation of active cells regarding their metabolic reduction potency.

Seven concentrations of phyco-synthesized MgO-NPs (7.8, 15.6, 31.25, 62.5, 125, 250, and 500 μg mL^−1^) were prepared to study their effect on the viability of Caco-2 and Vero cell lines within 48 h. MgO-NPs exhibited cytotoxic effects against normal and cancer cell lines in a dose-dependent manner. After the incubation with MgO-NPs for 48 h, the cloned cells suffer a partial or complete breakdown of the characteristic integral monolayer of epithelial cells along with shrinkage, buoyancy and the cells appear grainy and spherical. Besides the changes that occurred in the typical epithelial morphology of the cell lines, there is a decrease in the cells population. These cellular modifications can be explained by the entry of MgO-NPs into mammalian cells *via* endocytosis or macro-pinocytosis followed by enhanced synthesis of reactive oxygen species (ROS) that damage the membrane mitochondrial potential and lead to the activation of the apoptotic pathway, which ends with cell death (Verma et al., 2020)**.** Furthermore, the cellular damage induced by MgO-NPs is size-dependent, smaller-sized MgO NPs boosted the production of ROS, enhanced the interaction with cellular components, and improved membrane permeation to liberate Mg^+^ ions (Ratan et al., 2020). Likewise, Pugazhendhi et al. have reported the cytotoxic potential of MgO-NPs against lung cancer cell lines (A549), and observed cell rounding and shrinkage, membrane blabbing, apoptotic body formation, chromatin condensation, and reduced cell population after cells were incubated with MgO-NPs (Pugazhendhi et al., 2019).

**FIGURE 8:**
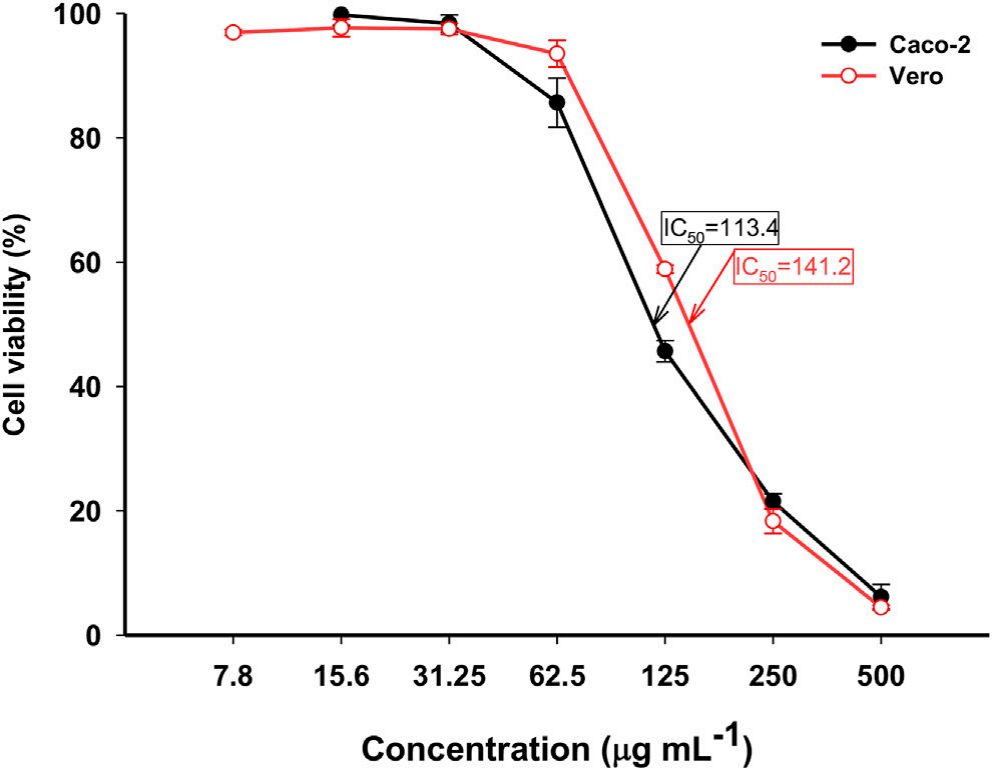
The cytotoxic activity of MgO-NPs derived from *Cystoseira crinita* against normal (Vero) and cancer (Caco-2) cell lines.

Our results for the *In-Vitro* cytotoxicity assay demonstrated the dramatic effect of MgO-NPs on the viability of treated cell lines and increasing the concentration of MgO-NPs has significantly reduced cell viability **(** Figure 8). However, the biocolloidal MgO reduced the viability of cancer, as well as normal cell lines. The IC_50_ concentration against the Caco-2 cancer cell line was 113.4, whereas it was 141.2 μg mL^−1^ for the normal Vero cell line. This threshold can be exploited clinically for selective targeting of cancer treatment. Accordingly, we claimed that MgO-NPs are more toxic to the cancer cell line than to the healthy cell line. Comparable cytotoxicity analysis concluded the enhanced anticancer potential of green synthesized MgO-NPs against cancer cell line compared to the normal cell line (Behzadi et al., 2018; Pugazhendhi et al., 2019; Amina et al., 2020)**.**

### Mosquitocidal Bioassay

#### Larvicidal and Pupicidal Activity

Regardless of their annoying habits, more than humans and animals’ pathogens are transmitted by physical association with house flies and it has been accused of transmitting many diseases such as cholera, infantile diarrhea, dysentery, typhoid fever, and an assortment of parasitic worms (Dahlem, 2009). Because they are closely related to humans, *Musca domestica* and its microbiome are of worth for detailed research (De Jonge et al., 2020). Therefore, we evaluated the larvicidal and pupicidal activities of phyco-synthesized MgO-NPs, in addition to their repellent properties.

The results listed in Table 1 manifested the effect of dipping the first, second, and third instar larvae and pupae of *M. domestica* in different concentrations (2, 4, 6, 8, and 10 μg mL^−1^) of phyco-synthesized MgO-NPs. The MgO-NPs caused larval death in a dose-dependent manner, and the mortality for first-stage larvae was 40 and 99% for concentrations of 2 and 10 μg mL^−1^, respectively. In contrast the mortality for second-stage larvae was 36.6 and 95% when treated with 2 and 10 μg mL^−1^ of MgO-NPs, respectively. Third instar larvae recorded a death rate of 30.8 and 92.2% corresponding to 2 and 10 μg mL^−1^ of MgO-NPs, respectively. Porbit program analysis calculated the LC_50_ concentrations by 3.08, 3.49, and 4.46 μg mL^−1^ and the LC_90_ concentrations by 7.46, 8.89, and 10.4 μg mL^−1^ for the first, second, and third instar larvae, respectively (Table 1).

**TABLE 1:** Larval and pupal toxicity of the phyco-synthesized MgO-NPs against the house fly *Musca domestica*.

Targeted instars	Mortality percentages (%) at different MgO-NPs concentrations					LC_50_	LC_90_
	2 μg mL^−1^	4 μg mL^−1^	6 μg mL^−1^	8 μg mL^−1^	10 μg mL^−1^		
I	40.0 ± 2.54	61.6 ± 3.78	73.2 ± 2.58	94.4 ± 1.14	99.0 ± 1.22	3.08	7.46
II	36.6 ± 2.30	57.2 ± 1.48	67.6 ± 1.81	86.2 ± 2.58	95.0 ± 1.73	3.49	8.89
III	30.8 ± 2.38	42.4 ± 2.50	61.2 ± 3.42	72.2 ± 3.11	92.2 ± 2.58	4.64	10.4
Pupa	22.4 ± 2.60	36.8 ± 1.64	51.0 ± 2.64	63.4 ± 2.96	81.0 ± 3.16	5.86	12.3

Mortality averages are means ± SD, of five replicates. No mortality was exhibited in the control. LC_50_ is the lethal concentration that kills 50%. LC_90_ is the lethal concentration that kills 90% of the treated larva or pupa.

Our specific feeding test demonstrated the potent toxicity of the phyco-synthesized MgO-NPs complex against the pupal phase of houseflies, 2 μg mL^−1^ of MgO-NPs inhibited pupation by 22.4%, and the maximum concentration of colloidal MgO solution (10 μg mL^−1^) inhibited pupation by 81%. The concentrations of LC_50_ and LC_90_ against the pupal phase were estimated to be 5.86 and 12.3 μg mL^−1^, respectively. Hence, we established the highly toxic effect of phyco-synthesized MgO-NPs against the larval and pupal stages of the housefly in a dose-dependent manner. Moreover, our results indicated that the phyco-synthesized MgO-NPs were more toxic to larvae than to pupae (Table 1).

Similarly, Fouda and coauthors reported the potency of the bio-synthesized MgO-NPs against different pupal and larvae instar of *Anopheles stephensi*, with LC_50_ values at 16.5 μg mL^−1^ for pupa and 12.5–15.5 μg mL^−1^ for I–IV larvae instar (Fouda et al., 2021a). Adesuji and coworkers reported the larvicidal efficiency of biosynthesized Ag-NPs against different instar larva phases of *Culex quinquefasciatus* (vector of lymphatic filariasis), recording the LC_50_ and LC_90_ values of 4.43 and 8.37 μg mL^−1^, respectively (Adesuji et al., 2016). In the same context, *Ambrosia arborescens* leaf extracts and Ag-NPs derived from these extracts proved larvicidal properties against the third-stage larvae of *Aedes aegypti* mosquitoes (the main vector of Zina, dengue, and chikungunya infections). Moreover, Ag-NPs were more toxic than the plant extracts and scored the LC_50_ at 0.28 μg mL^−1^, LC_90_ at 0.43 μg mL^−1^, whereas the aqueous extracts showed the massive values of LC_50_ and LC_90_ of 1844.61 and 6043.95 μg mL^−1^, respectively (Morejón et al., 2018).

The mosquitocidal activities of MgO-NPs may be mainly attributed to their effectiveness in producing the elevated amount of ROS compared with other metal oxide nanoparticles, while being low toxic to plants, animals, and humans, besides their ability to damage the cell wall (Krishnamoorthy et al., 2012; Ma et al., 2018). Moreover, the increased Mg^2+^ concentration disturbs cellular equilibrium causing leakage of cell ingredients that end with mosquito cellular death (Pugazhendhi et al., 2019). The histological response to biogenic Ag-NPs reported that brush border and epithelial cells of the midgut area of the larvae of *Aedes albopictus* were highly affected (Ga’al et al., 2018). Moreover, metallic nanoparticles might bind to P and S in nucleic acids and proteins, respectively, which leads to the reduced permeability of the membrane, denaturation of enzymes and organelles ending in cell death. In addition, metal and metal oxide nanoparticles regulate important genes of insects, decrease releasing of gonadotrophin and protein synthesis, which leads to reproductive failure and damage to growth (Benelli, 2018). Based on these results, the phyco-synthesized MgO-NPs could be introduced as an efficient candidate for the biological control of mosquitoes.

#### Repellent Activity

Arthropods land on the skin and bite, which can be a carrier of some diseases. Repellents are chemicals that prohibit arthropods from landing on the skin and thus prevent bites and the resulting disease transmission (Tavares et al., 2018). Although synthetic insecticides and repellents are easy to use and fast-acting, their continued use disrupts the ecological balance and the consequent damage to nontarget organisms, as well as the development of insect resistance, in addition to the cost of these chemicals limiting their use in low-income countries (Fernandes et al., 2021). As research continues to create economical and environmentally safe alternatives that have insecticidal properties, in this research, we explored the properties of phyco-synthesized MgO-NPs as insect repellent. Here we set a time-based bioassay to explore the mosquito-repellent properties of the phyco-synthesized MgO-NPs against adult domestic mosquitoes and recorded observations after 12, 24, 48, and 72 h of incubation. The results confirmed the properties of phyco-synthesized MgO-NPs as a mosquito repellent in a time-dependent manner.

The experiment was conducted using 1.0% MgO-NPs at a concentration of 10 μg mL^−1^. After 12 h from the beginning of the test, the repellent rate was 63 ± 0.23%, whereas the repellency percentages after 24, 48, 72 h were increased to be 77.9 ± 0.34, 84.9 ± 0.31, and 96.8 ± 0.213%, respectively. In the same regard, Hassan and coauthors studied the insecticidal properties of the myco-synthesized MgO-NPs against *Culex pipiens* and they reported the larvicidal effect and repellent potency of quite low concentrations of MgO-NPs against the common house mosquito (Hassan et al., 2021). Recently, the mosquito-repellent efficiency of Phyto-derived Ag-NPs was confirmed against four-vector mosquitoes; different concentrations of Ag-NPs (50, 75, and 100 mg mL^−1^) manifested 6.25–60.0% repellences against week-old female mosquitoes of *Culex quinquefasciatus*, *Anopheles gambiae*, *Anopheles maculatus*, and *Aedes aegypti* (Akintelu et al., 2021). Likewise, a recently published study concluded the successful fabrication of MgO-NPs through harnessing metabolites of *Penicillium chrysogenum* and exhibited the broad-spectrum antibacterial activity, larvicidal, and pupicidal activity coupled with long-lasting mosquito-repellent properties against adults of *Anopheles stephensi* (Fouda et al., 2021a). As the phyco-synthesized MgO-NPs obtained in the current study are one-step easy synthesized, inexpensive, and attain several biological properties, we recommend exploiting them in medicinal applications as well as in the designing of topical mosquito repellents to control mosquito-borne diseases, especially in endemic countries.

### Comparison Study

The efficacy of *Cystoseria* spp. to fabricate different metal and metal oxides nanoparticles with varied shapes, sizes, and applications compared with the current study is shown in Table 2. As shown, the polysaccharide fucoidan secreted by *Cystoseira barbata* have the efficacy to fabricate spherical Ag-NPs and Se-NPs with sizes of 12.86 and 16.18 nm, respectively, and showed anti-*Candida* activity (Alghuthaymi et al., 2021). Moreover, the spherical CuO-NPs with the size of 11–80 nm were successfully synthesized by the aqueous extract of *Cystoseira myrica* and exhibit high activity against two cancerous cell lines, namely, HepG2 and MCF-7 (Mohamed et al., 2021). In a recent study, the aqueous extract of *C. crinita* was used to form rectangular ZnO-NPs with sizes ranging between 23 and 200 nm and showed antimicrobial and antioxidant activity (Elrefaey et al., 2022). To the best of our knowledge, this is the first report for the green synthesis of MgO-NPs using an aqueous extract of *C. crinita*. In the current study, a small size (3–18 nm) of spherical MgO-NPs was successfully formed by brown algae, *C. crinita,* and exhibited varied activity including antimicrobial, *in-vitro* cytotoxicity, larvicidal, and repellence activity.

**TABLE 2:** The efficacy of *Cystoseira* spp. to fabricate metal and metal oxides nanoparticles and compare them with the current study.

*Cystoseira spp.*	NPs	Characterized by	Shape and size	Applications	Ref
*Cystoseira baccata*	Au-NPs	UV-vis spectroscopy, TEM, and zeta potential	Spherical shape with a size of 8.4 nm	*In-vitro* cytotoxicity against normal and cancer cell lines	González-Ballesteros et al. (2017)
C. myrica	CuO-NPs	UV-Vis spectroscopy, TEM, DLS, XRD, and FTIR.	Spherical shape with a size of 11–80 nm	*In-Vitro* cytotoxicity	Mohamed et al. (2021)
*C. crinita*	ZnO-NPs	UV-Vis spectroscopy, TEM, FT-IR, XRD, and DLS	Multilayered rectangular particles with sizes of 23–200 nm	Antimicrobial and antioxidant	Elrefaey et al. (2022)
*C. barbata*	Ag-NPs	UV-Vis spectroscopy, FT-IR, DLS, and TEM	Spherical shape with a size of 12.86	Anti-*Candida*	Alghuthaymi et al. (2021)
*C. barbata*	Se-NPs	UV-Vis. FT-IR, DLS, and TEM	Spherical shape with a size of 12.86	Anti-*Candida*	Alghuthaymi et al. (2021)
*C. crinita*	MgO-NPs	UV-Vis, FT-IR, XRD, SEM-EDX, TEM, and XPS	Spherical shape with size of 3–18 nm	Antimicrobial, *in-vitro* cytotoxicity, larvicidal, and repellence activity	Current study

## Conclusion

In the current study, the aqueous extract of *C. crinita* was used as a reducing and capping/stabilizing agent for the green synthesis of MgO-NPs. The color change of algal aqueous extract from pale yellow to yellowish-brown as well as observing the maximum SPR peak at 320 nm confirmed the MgO-NPs formation. The crystalline structure and spherical shape with sizes of 3–18 nm were investigated by XRD and TEM analyses. Also, the SEM-EDX and XPS revealed that the main components of the resultant are Mg and O. The activities of algal-mediated green synthesis of MgO-NPs were dose-dependent. The synthesized MgO-NPs showed excellent antimicrobial activity against various pathogenic bacteria, and unicellular fungi. Data showed that the synthesized MgO-NPs showed high selectivity to destroy cancer cell lines at low concentrations compared with normal cell lines. The biosynthesized MgO-NPs showed larvicidal/pupicidal of *M. domestica* at low concentration against I, II, III instar larvae, and pupa. Moreover, it exhibited a repellence activity against adults of *M. domestica* with percentages of 63 ± 0.23%, 77.9 ± 0.34%, 84.9 ± 0.31%, and 96.8 ± 0.213% after 12, 24, 48, and 72 h, respectively. The obtained data confirm the power of active substances produced by brown algae, *C. crinita* to fabricate MgO-NPs that are characterized by their activity as well as biocompatibility into various biomedical applications.

## Data Availability

The data used to support the findings of this study are available from the corresponding author upon request.
